# Recombination Drives Evolution of Carbapenem-Resistant Klebsiella pneumoniae Sequence Type 11 KL47 to KL64 in China

**DOI:** 10.1128/spectrum.01107-22

**Published:** 2023-01-09

**Authors:** Tao Chen, Yuan Wang, Yanzi Zhou, Wangxiao Zhou, Xiaohui Chi, Ping Shen, Beiwen Zheng, Yonghong Xiao

**Affiliations:** a State Key Laboratory for Diagnosis and Treatment of Infectious Diseases, National Clinical Research Center for Infectious Diseases, Collaborative Innovation Center for Diagnosis and Treatment of Infectious Diseases, the First Affiliated Hospital, Zhejiang University School of Medicine, Hangzhou, Zhejiang, China; b Jinan Microecological Biomedicine Shandong Laboratory, Jinan, Shandong, China; National Taiwan University

**Keywords:** *Klebsiella pneumoniae*, ST11, recombination, evolution, capsule, lipopolysaccharide

## Abstract

Carbapenem-resistant *Enterobacteriaceae*, especially carbapenemase-producing Klebsiella pneumoniae, is an urgent problem in health care facilities worldwide. K. pneumoniae isolates classified as sequence type 11 (ST11) are largely responsible for the spread of carbapenem-resistant K. pneumoniae (CRKP) in China. Our previous phylogenetic reconstruction suggested that CRKP ST11 capsular locus 64 (KL64) was derived from an ST11-KL47 ancestor through recombination. However, the molecular origin of KL64 remains largely unknown, and our understanding of the recombination is incomplete. Here, we screened a global sample of 22,600 K. pneumoniae genomes and searched for KL64-harboring STs, which were found to be ST1764, ST3685, ST1764-1LV, ST30, ST505, ST147, and ST11, wherein ST1764, ST3685, ST1764-1LV, and ST30 belonged to a clonal complex, CC1764. We compared the genetic structures of representative strains from ST11-KL47, ST11-KL64, CC1764-KL64, ST505-KL64, and ST147-KL64 and further performed phylogenetic analysis and single-nucleotide polymorphism analysis among 248 isolates from all these STs. The results suggested a recombination event has occurred in a homologous ~154-kb region covering KL and the lipopolysaccharide biosynthesis locus (OL) between a recipient ST11-KL47-OL101 and a donor CC1764 (except ST30), giving rise to ST11-KL64-O2v1 strains (recombination I). Furthermore, we also found an infrequent ST11-KL64-O2v1 subclone which was not produced by recombination I but was hybridized from ST11-KL47-OL101 and ST147-KL64-O2v1 strains through recombination of a homologous ~485-kb region covering KL and OL (recombination II). Our findings provide important insights into the role of recombination in the evolution of clinical strains and the diversity of capsule and lipopolysaccharide of widely distributed KPC-associated ST11 K. pneumoniae in China.

**IMPORTANCE** Chromosomal recombination events are considered to contribute to the genetic diversification and ultimate success of many bacterial pathogens. A previous study unravelled the molecular evolution history of ST258 strains, which have been largely responsible for the spread of KPC in the United States. Here, we used comparative genomic analyses to discover two recombination events in ST11 CRKP strains, which is a predominant KPC-associated CRKP clone in China. Two new ST11-CRKP subclones with altered capsule and lipopolysaccharide, which are two primary determinants of antigenicity and antigenic diversity among K. pneumoniae, were produced through these two recombination events, respectively. Horizontal transfer of the KL and OL appears to be a crucial element driving the molecular evolution of K. pneumoniae strains. These findings not only extend our understanding of the molecular evolutionary history of ST11 but also are an important step toward the development of preventive, diagnostic, and therapeutic strategies for CRKP.

## INTRODUCTION

In recent decades, carbapenem-resistant *Enterobacteriaceae* (CRE) have become an urgent public health concern worldwide with few treatment options. Among these, carbapenem-resistant Klebsiella pneumoniae (CRKP) strains account for more than half of clinical CRE infections in the European Union, United States, and China (1–3). In the United States, CRKP sequence type 258 (allelic profile 3-3-1-1-1-1-79) emerged as a notable clinical problem during the early to mid-2000s and remains the main ST in the United States and elsewhere (4, 5). Phylogenetic analysis of the core genome of ST258 K. pneumoniae strains revealed that they are comprised of two distinct genetic clades (ST258 clades I and II), largely due to an ~215-kb region of divergence that includes genes involved in capsular polysaccharide (CPS) biosynthesis (6). Furthermore, ST258 is thought to be a hybrid clone caused by a recombination event—80% of the genome originated from ST11-like strains and 20% from ST442-like strains, and the ST258 clade I strains evolved from a clade II strain as a result of the replacement of an ~52-kb region encoding the CPS biosynthetic machinery (7). Extensive capsule locus variation is believed to be primarily responsible for clonal group 258 (CG258) diversification, and recombination spanning the CPS locus is considered a crucial mechanism by which lineages diversify their antigenic profile against exterior selection pressures (8, 9). These studies consistently indicated that the genomic region involved in CPS biosynthesis in Klebsiella pneumoniae is a vital recombination hot spot.

In China, ST11 (allelic profile 3-3-1-1-1-1-4), a single-locus (*tonB*) variant of ST258, is believed to be the most dominant Klebsiella pneumoniae carbapenemase (KPC)-associated CRKP clone, with a prevalence greater than 60% (10). Currently, more concerning is the emergence of carbapenem-resistant hypervirulent Klebsiella pneumoniae (CR-hvKP), which may lead to even higher mortality and morbidity (11). Of note, several interhospital outbreaks of ST11 CR-hvKP with capsular locus 64 (KL64) and plasmid-encoded virulence genes were reported recently (12, 13). Among ST11 CR-hvKP in China, the group of KL64 strains is the most widespread, as it has been observed to harbor more plasmid-encoded virulence genes than ST11 non-KL64 strains (14–16). Our previous study found a subclonal shift in the dominant clone ST11 CRKP in which the previously prevalent KL47 had been replaced by KL64 and enhanced virulence of ST11-KL64 CRKP was found by phenotypic tests (17). By using a Bayesian dating method, we found that KL64 isolates probably evolved from a KL47 ancestor, but the molecular donor of KL64 and the hypothetical recombination event were not further explored.

In this study, we sought to find the molecular origin of KL64 and a probable recombination event leading to the capsular switching from KL47 to KL64 among CRKP-ST11 populations in China. In addition, we discovered a special infrequent ST11-KL64 subclone, different from the predominant ST11-KL64 subclone, which might have been produced by another recombination event.

## RESULTS

### An ~154-kb genomic region covering KL and OL was distinctly different between ST11-KL47 and ST11-KL64 K. pneumoniae.

The genomes of two ST11 K. pneumoniae clinical isolates (KP16932 and KP47434) were obtained from a hybrid assembly (GenBank accession numbers QVAN00000000 [KP16932] and QURI00000000 [KP47434]). The result of genomic analysis showed KP16932 contained CPS biosynthesis locus (K_locus [KL]) KL47 and LPS biosynthesis locus (O_locus [OL]) OL101, while KP47434 contained KL64 and O2v1. We took these two complete genomes as representative genomes for ST11-KL47 and ST11-KL64 isolates, respectively. As KL is close to OL in genomic location, further analysis of the KL, OL, and flanking nucleotide sequences revealed that the differences between ST11-KL47 and ST11-KL64 are expansive, covering an ~154-kb contiguous region corresponding to nucleotide positions ~4,060,800 to ~4,214,550 in strain KP47434. This ~154-kb contiguous region covers the ~52-kb region of which the replacement was believed to cause the evolution of ST258 clade I strains from a clade II strain (7). The ~154-kb region contained 129 open reading frames (ORFs), such as KL, OL, the *hisLGDCBHAFI* operon (which encodes the enzymes of the histidine biosynthesis pathway), *mdtABCDQ* multidrug transporters, the two-component system BaeSR (involved in envelope stress response), and so on. All ORFs annotated by the RAST server in the ~154-kb region are listed in Table S3 in the supplemental material. We carefully compared the ~154-kb contiguous regions among these isolates. Analysis of single-nucleotide polymorphisms (SNPs) in the genomes of KP16932 and KP47434 indicated that these strains differed by 1,233 SNPs, of which 903 SNPs (~73%) were concentrated in the contiguous ~154-kb region. Furthermore, 79% coverage and 97.94% identities were found in the ~154-kb contiguous region between KP16932 and KP47434. According to these results in combination with the previous results from the Bayesian dating method, which indicated KL64 isolates probably evolved from a KL47 ancestor (17), we speculated that the ~154-kb region covering KL and OL was a recombination region acquired from other unrelated STs by ST11-KL47 to evolve into ST11-KL64.

### CPS biosynthesis and LPS biosynthesis loci of K. pneumoniae isolates belong to different STs.

**FIG 1:**
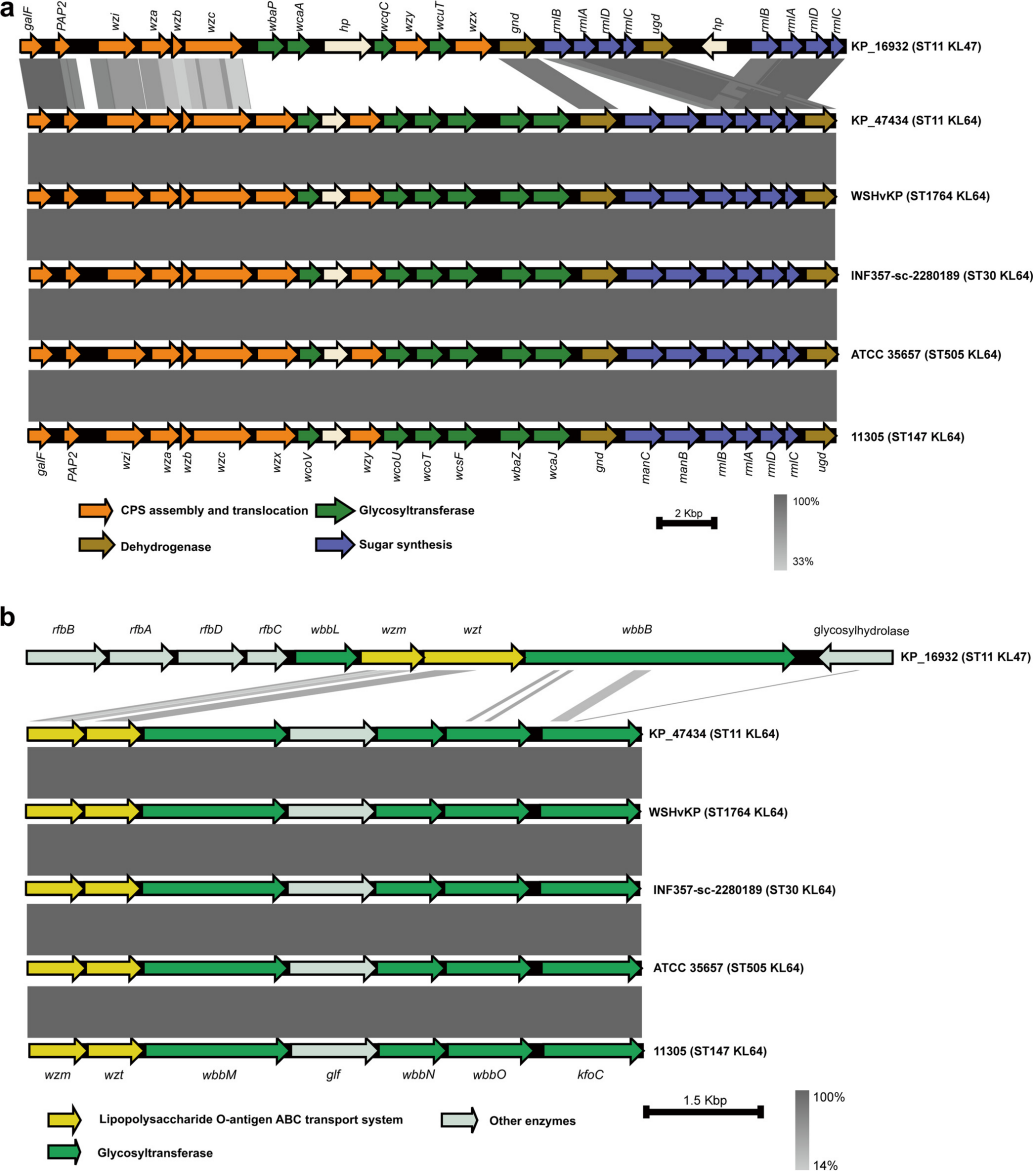
Comparison of the KL (a) and OL (b) in strains KP16932 (ST11-KL47), KP47434 (ST11-KL64), WSHvKp (ST1764-KL64), INF357-sc-2280189 (ST30-KL64), ATCC 35657 (ST505-KL64), and 11305 (ST147-KL64). Arrows indicate the direction, relative length, and function of ORFs. Homologous regions are connected by areas of different colors reflecting the degree of nucleotide identity. Sequence comparisons were performed using BLASTn and visualized using Easyfig.

To seek the possible molecular origin of recombination region, we selected K. pneumoniae strains of different STs which harbored KL64 genetic locus from 22,600 genomes as described in Materials and Methods. We found that KL64 could be detected in the genomes of ST1764 (allelic profile 5-3-1-1-9-4-283), ST3685 (5-3-1-232-9-4-283), ST1764-1LV (5-3-1-1-9-4-683), ST30 (5-3-1-1-9-4-26), ST505 (7-1-5-1-1-1-84), ST147 (3-4-6-1-7-4-38), and ST11 (3-3-1-1-1-1-4). Among these, ST1764, ST3685, ST1764-1LV, and ST30 belonged to a clonal complex, CC1764. A complete genome was selected as a representative genome for each ST (or CC): strain WSHvKp (GenBank accession number GCA_013283935.1) for CC1764 except ST30, strain INF357-sc-2280189 (GenBank accession number GCA_904863125.1) for ST30, strain ATCC35657 (GenBank accession number GCA_001936035.1) for ST505, and strain 11305 (GenBank accession number GCA_009931095.1) for ST147. The results of genomic analysis showed that strains WSHvKP, INF357-sc-2280189, and ATCC 35657 contained the same KL and OL (KL64 and O1v1), while strain 11305 harbored KL64 and O2v1. We further compared the nucleotide sequences of the KL and OL in these isolates by using BLAST. As shown in Fig. 1, KP47434, WSHvKP, INF357-sc-2280189, ATCC 35657, and 11305 displayed almost identical nucleotide sequences for KL and OL which were distinctly different from those of KP16932.

### The ~154-kb recombination region covering KL and OL in ST11-KL64 was derived from CC1764 (except ST30).

To find the possible ST from which the recombination region originated, we compared the contiguous ~154-kb regions among these representative isolates. Interestingly, the ~154-kb regions were nearly identical in KP47434 and WSHvKP, differing by only 8 SNPs, while the genomes of KP47434 and WSHvKP differed by 22,407 SNPs in total. By comparison, the SNP distances between KP47434 and INF357-sc-2280189, ATCC 35657, and 11305 in this region were 35, 720, and 770 SNPs, respectively. Furthermore, 99% coverage and almost 100% identities were found in this region between KP47434 and WSHvKP, indicating the ~154-kb region in ST11-KL64 strains was more closely related to strains of ST1764 than ST30, ST505, and ST147. These results suggested that the ~154-kb region in ST11-KL64 strains might have been derived from CC1764 (except ST30), which led to an assumption that a recombination event (recombination I) probably has occurred in a homologous ~154-kb region covering KL and OL between a recipient ST11-KL47-OL101 and a donor CC1764 (except ST30), giving rise to ST11-KL64-O2v1.

**FIG 2:**
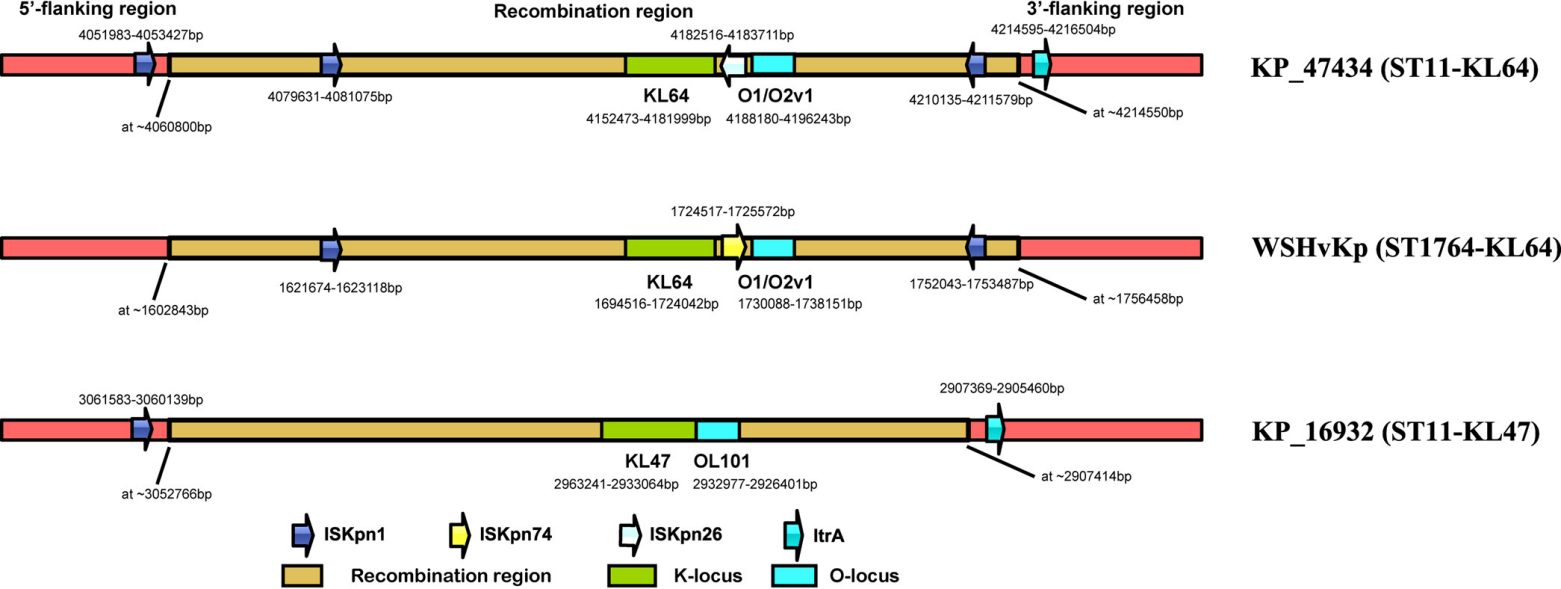
Mobile genetic elements analysis of the contiguous ~154-kb region and flanking sequences in KP16932 (ST11-KL47), KP47434 (ST11-KL64), and WSHvKP (ST1764-KL64) strains. Arrows indicate the direction of an IS. Different colors reflect the different kinds of ISs. The recombination region, KL, and OL are shown in different colors. The image was not drawn to scale.

To provide further evidence to the speculation, we performed comparative sequence analysis focusing primarily on mobile genetic elements of the contiguous ~154-kb region and flanking sequences. We found an ISKpn1 (nucleotide positions 4,051,983 to 4,053,427 in strain KP47434) and an ltrA (nucleotide positions 4,214,595 to 4,216,504 in strain KP47434) encoding group II intron reverse transcriptase or maturase at the neighboring sequences upstream and downstream from this ~154-kb region in ST11 strains (KP16932 and KP47434), respectively. As expected, they were absent in ST1764 strain WSHvKP (Fig. 2). Both of these findings indicated the ISKpn1 and *ltrA* were probably not a part of the recombination region. Moreover, inside the ~154-kb recombination region we assumed, two ISKpn1 (nucleotide positions 4,079,631 to 4,081,075 and 4,210,135 to 4,211,579 in strain KP47434) at the identical position with the same orientation were found in KP47434 and WSHvKP, which were absent in KP16932 (Fig. 2). All these findings provided further support for the assumed recombination event. Unexpectedly, inside the ~154-kb recombination region, an ISKpn26 was observed in KP47434 (nucleotide positions 4,182,516 to 4,183,711 in strain KP47434) but an ISKpn74 (nucleotide positions 1,724,517 to 1,725,572 in strain WSHvKP) was found in WSHvKP at the same genomic location. Therefore, we supposed an insertion sequence (IS) substitution had occurred after the ~154-kb recombination event.

**FIG 3:**
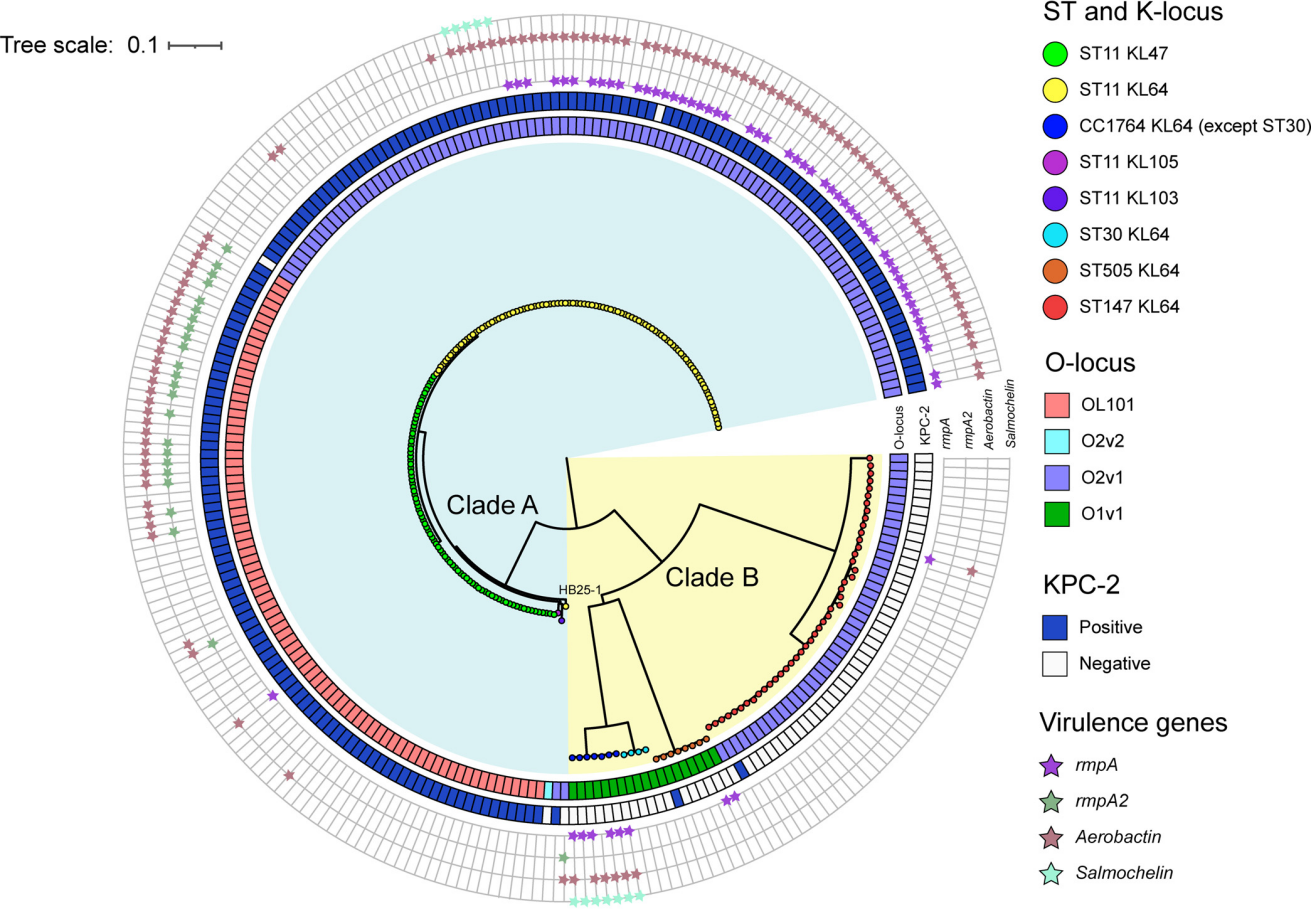
Genomic core SNP-based phylogenetic analysis of 248 Klebsiella pneumoniae strains, showing branch lengths. Colored rectangles indicate, from the innermost to the outermost, the lipopolysaccharide biosynthesis locus and presence of the *bla*_KPC-2_ gene. The presence of virulence genes is indicated by small pentacles of diverse colors.

**FIG 4:**
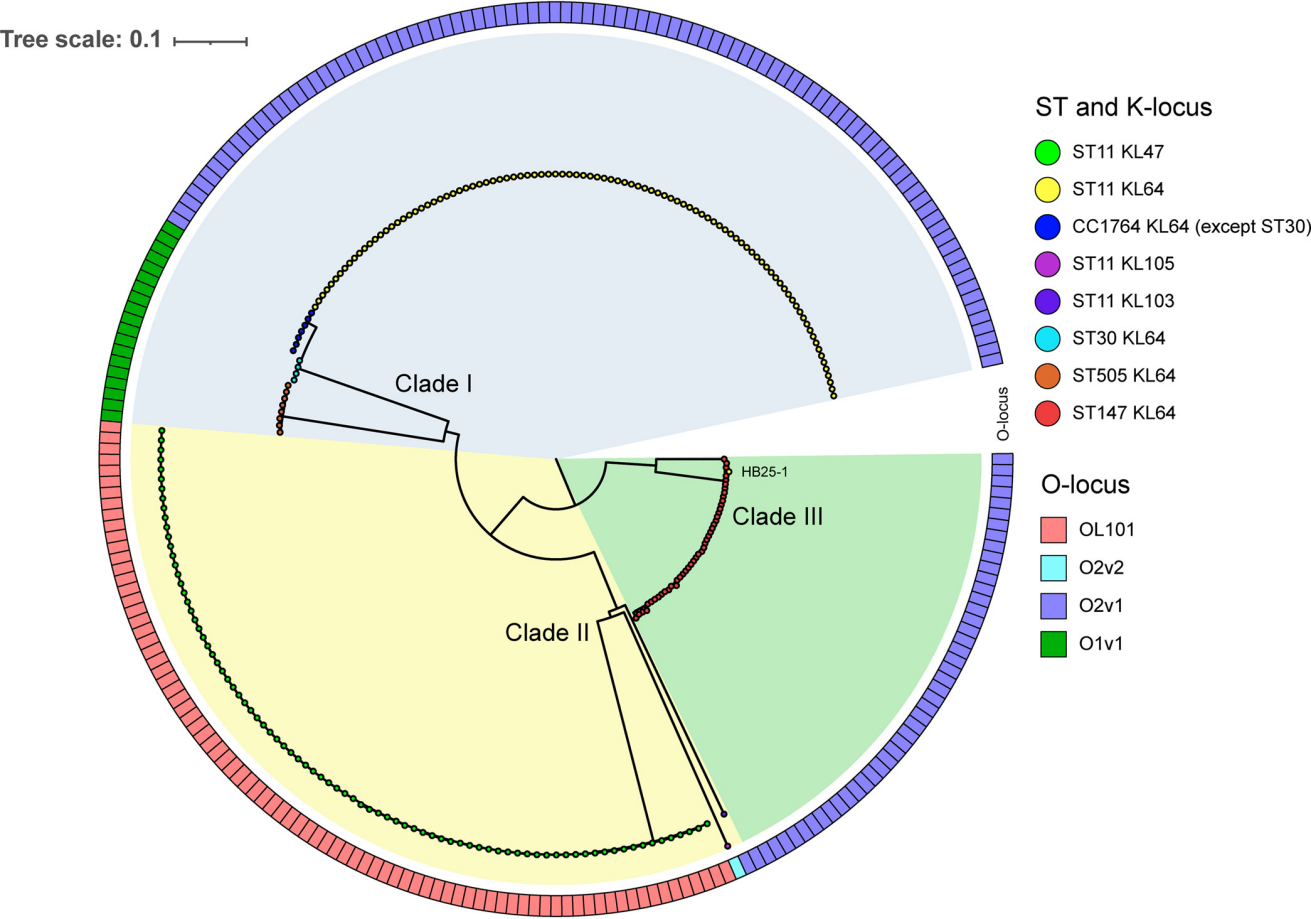
Phylogenetic analysis of 248 Klebsiella pneumoniae strains based on core SNPs located in the ~154-kb region. Different STs and KLs are marked with different colors. Rectangles in different colors indicate different lipopolysaccharide biosynthesis loci.

To analyze the hypothetical recombination region at the level of multiple isolates, we performed a phylogenetic analysis, including all of the 248 K. pneumoniae genomes used in this work, on the genomic core SNPs and in parallel on the core SNPs located in the ~154-kb region. The phylogenetic tree of genomic core SNPs showed the 248 strains were clustered into two major groups, which were named clade A and clade B and contained 184 and 64 strains, respectively (Fig. 3 and Fig. S1). All strains in clade A belonged to ST11 with various KL and OL (including KL47-OL101, KL64-O2v1, KL103-O2v1, and KL105-O2v2), while all strains in clade B were other STs (CC1764-KL64-O1v1, ST505-KL64-O1v1, and ST147-KL64-O2v1). Almost all ST11 strains in clade A produced the well-known KPC-2 carbapenemase, while other STs did not. Interestingly, ST11-KL64 strain HB25-1, identified in the Second Affiliated Hospital of Zhejiang University in China (18), was not grouped with other ST11-KL64 strains in clade A, which suggested the emergence of a special ST11-KL64 subclone. In contrast, the resulting phylogenetic tree of the core SNPs located in the ~154-kb region demonstrated that 248 strains were clustered into three major groups, which were named clade I, clade II, and clade III, and contained 116, 86, and 46 strains, respectively (Fig. 4 and Fig. S1). The strains in clade I comprised ST11-KL64, CC1764-KL64, and ST505-KL64 strains. Moreover, CC1764-KL64 (except ST30) strains were more closely related to ST11-KL64 strains than ST30-KL64 and ST505-KL64 strains in clade I. The strains in clade II comprised strains of ST11-KL47, ST11-KL103, and ST11-KL105, while clade III contained all ST147-KL64 strains and the ST11-KL64 strain HB25-1. These results suggested that the ~154-kb region in ST11-KL64 strains (except strain HB25-1) was more closely related to strains of CC1764 (except ST30) than ST30, ST505, and ST147, providing further support to the assumed recombination event. Notably, the ST11-KL64 strain HB25-1 was grouped with ST147-KL64 strains in clade III (see below).

Minimum spanning trees (MSTs) were also generated based on SNP data for the genomes and the ~154-kb regions of 248 strains. The genomic core SNP distances of isolates of different STs suggested that the isolates from different groups were distinctly related and isolates within the same group were highly related (Fig. S2). The ST11-KL64 group showed a significantly closer SNP distance from ST11-KL47 than other STs (only 1,099 SNPs between the central strains of ST11-KL64 and ST11-KL47). However, it should be noted that strain HB25-1 showed a distance of 2,191 SNPs from the central strain of ST11-KL64. By comparison, the core SNP distances within the ~154-kb region of isolates of different STs (Fig. S3) suggested the ~154-kb region in ST11-KL64 group (except strain HB25-1) was more closely related to CC1764 (except ST30) than ST30, ST11-KL47, ST505, and ST147 (only 2 SNPs between the central strains of ST11-KL64 and CC1764 except ST30). All these findings indicated that the donor of the ~154-kb region in ST11-KL64 was closely related to that for CC1764 isolates (except ST30), providing further support to the recombination hypothesis. Interestingly, the ST11-KL64 isolate HB25-1 showed a closer SNP distance to ST147-KL64 isolates in the MST based on the ~154-kb region, suggesting novel recombination events or evolutionary convergence were associated with this specific ST11-KL64 isolate.

**FIG 5:**
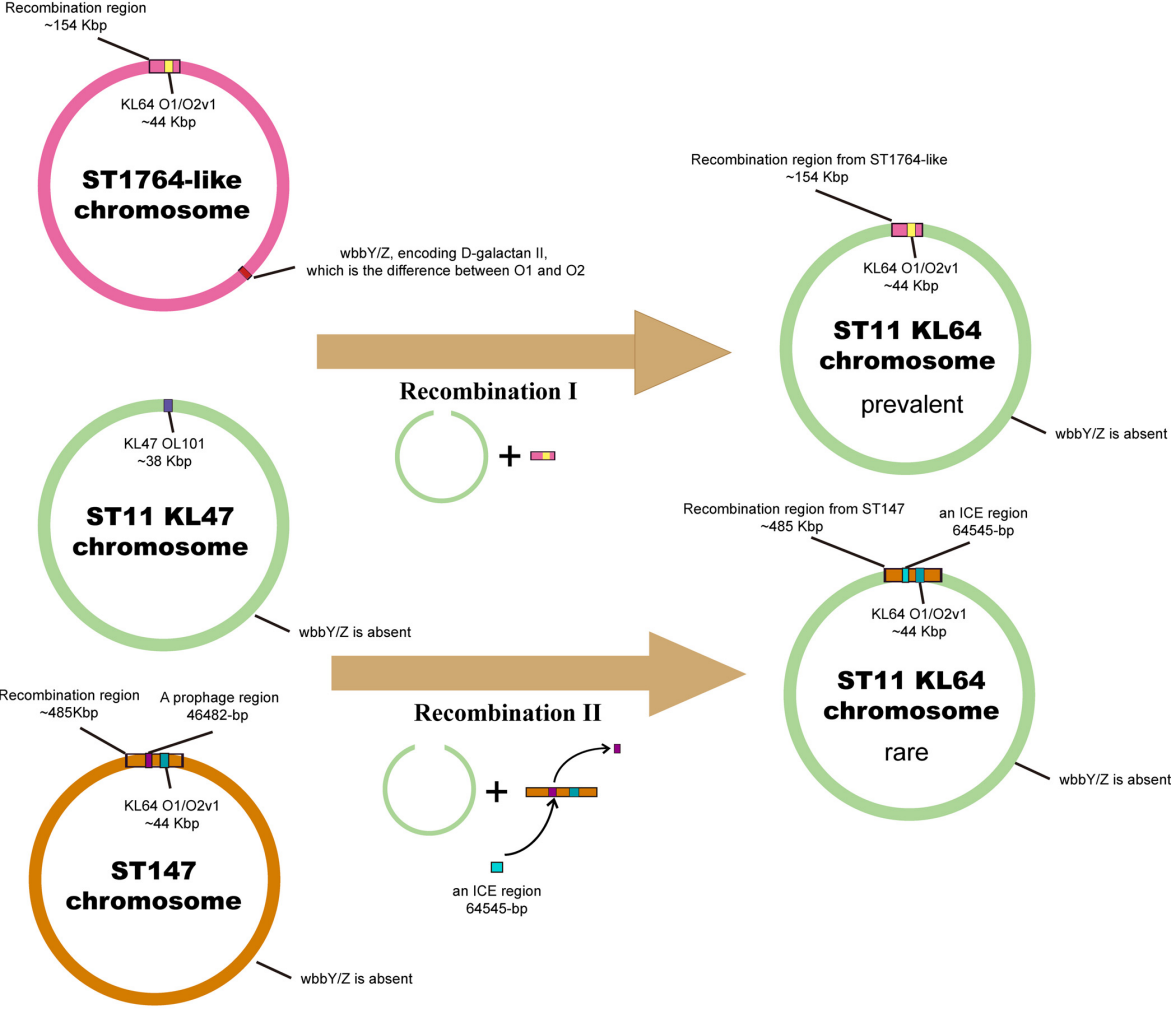
Two recombination events occurred in Klebsiella pneumoniae ST11 strains. Schematic representation based on the results of our analyses. Two recombination events (recombination I and recombination II) produced two ST11-KL64 descendants. The donors of KL64 and O2v1 among two ST11-KL64 subclones were CC1764 (except ST30) and ST147, respectively.

Given that ST11-KL64 is O2v1 and not O1v1 (as is CC1764-KL64), this aspect was at odds with our hypothesis. Thus, we performed a genomic analysis to find out what led to the different OL variants between ST11-KL64 and CC1764-KL64. It is known that both the O1 and O2 polysaccharide chains are based on a repeat unit named d-galactan I. The difference is that the O1 antigen is capped by a distal d-galactan II unit, whereas O2 is not. d-Galactan II is the only known O-antigen polysaccharide for which biosynthesis is enabled by genes (*wbbY* and *wbbZ*) located well away from the OL in Klebsiella genomes (19). As shown in Fig. 5, the ST11-KL47 chromosome lacks these genes (*wbbY* and *wbbZ*). Although the ~154-kb region covering OL in CC1764 (except ST30) was recombined to the chromosome of ST11-KL47, the OL of the recombination offspring could only directly transform from OL101 into O2 (as with ST11-KL64) but not O1 (as with CC1764-KL64) due to the absence of *wbbY* and wbb*Z*.

### HB25-1: a rare ST11-KL64 subclone with an ~485-kb contiguous region derived from ST147-KL64.

Because the ST11-KL64 isolate HB25-1 was not gathered with other ST11-KL64 strains in the phylogenetic tree that was based on the genomic core SNPs and gathered with ST147-KL64 strains based on the core SNPs located in the ~154-kb region, we speculated that another recombination event was associated with this specific isolate HB25-1. Likewise, we analyzed the KL, OL, and flanking nucleotide sequences to seek the differences between KP16932 and HB25-1, and an ~485-kb contiguous region corresponding to nucleotide positions ~1,676,640 to ~2,161,600 in strain HB25-1 was found to be significantly different. Analysis of SNPs in the genomes of KP16932 and HB25-1 indicated that these two strains differed by 3,001 SNPs, of which 2,079 SNPs (~69%) were concentrated in the contiguous ~485-kb region, implying that this region was probably a recombination region acquired from other unrelated STs. The RAST annotation results indicated that the ~485-kb region contained 456 ORFs, as shown in Table S4. Given the results that HB25-1 was gathered with ST147-KL64 strains based on the core SNPs located in the ~154-kb region (Fig. 4), we speculated that ST147-KL64 was the molecular donor of KL64 in strain HB25-1. We compared the SNP distances between HB25-1 and the ST147-KL64 strain 11305 in the ~485-kb region, and we found this region was nearly identical in HB25-1 and 11305, differing by only 26 SNPs. Furthermore, 90% coverage and 99.99% identities were found in this region between HB25-1 and 11305. The reason why the coverage was not nearly 100% was further found to primarily be the presence of mobile genetic elements, which were a 46,482-bp prophage (nucleotide positions 1,837,435 to 1,883,916) in strain 11305 and a 64,545-bp integrative and conjugative element (ICE; nucleotide positions 1,881,956 to 1,946,500) in strain HB25-1 (Fig. 5). These results indicated another recombination event (recombination II) probably occurred in a homologous ~485-kb region covering KL and OL between a recipient ST11-KL47-OL101 strain and a donor ST147-KL64-O2v1 strain, giving rise to a specific ST11-KL64-O2v1 subclone (like strain HB25-1) (Fig. 5).

To investigate the prevalence of the specific ST11-KL64 subclone like strain HB25-1, we performed a phylogenetic analysis of 623 genomes of ST11-KL64 K. pneumoniae strains isolated from China (including KP47434 and HB25-1) on the core SNPs located in the ~485-kb region. The results indicated only 1 of 621 genomes was grouped with strain HB25-1 in clade 1, while the other 620 genomes were grouped with KP47434 in clade 2 (see Fig. S4 in the supplemental material). These results suggested the ST11-KL64 subclones produced by recombination I were much more prevalent than those caused by recombination II in China.

## DISCUSSION

The current rise of KPC-producing K. pneumoniae infections in health care facilities in Asian countries has been overwhelmingly related to strains belonging to ST11, which is a single-locus variant of ST258 strains that are frequently found in the United States (20–22). Control of ST11 CRKP in populations and health care networks is often complicated due to rapid evolution. Therefore, it is crucial to track evolutionary events and understand their clinical importance. Previously, we found a KL shift (the previously prevalent KL47 had been replaced by KL64) in the dominant clone BSI-CRKP-ST11 over a 4-year period and that KL64 isolates probably evolved from a KL47 ancestor (17). In this work, we have provided independent evidence of the recombination events that drove evolution of widely distributed KPC-associated ST11 K. pneumoniae and resulted in capsule and LPS switching.

Recombination events and replacement of chromosomal regions have been documented in numerous bacteria, contributing to genetic plasticity, and the evolved strains are associated with epidemiological success. Staphylococcus aureus ST239, a pandemic methicillin-resistant S. aureus (MRSA) sequence type, is responsible for ~90% of the nosocomial infections throughout mainland Asia and South America (23). ST239 is believed to be comprised of large chromosomal regions from two distinctly related lineages, ST8 and ST30 (24). The replacement of approximately 20% of the ST8 genome by an ~550-kb contiguous chromosomal fragment from an ST30 donor strain generated the strains of novel sequence type ST239. In group B Streptococcus (GBS), genetic replacement of the CPS region between unrelated sequence types is the common mechanism by which this species alters its surface antigen composition, which contributes to the diversity of GBS and selection of the dominant clone (25).

Based on recent genome-scale studies on K. pneumoniae, there have been numerous putative chromosomal recombination events involving the region related to CPS biosynthesis (6, 26). Chen et al. illustrated ST258 clades I and II have distinct CPS regions and the ST258 clade I strains evolved from a clade II strain caused by the replacement of an ~52-kb region encoding the CPS biosynthetic machinery (7). Here, by using phylogenetic and SNP analyses, we discovered that a recombination event had occurred in a homologous ~154-kb region covering CPS and LPS biosynthesis loci between a recipient, ST11-KL47-OL101, and an unrelated donor from CC1764 (except ST30), giving rise to ST11-KL64-O2v1 (recombination I). This ~154-kb contiguous region covers the previously described ~52-kb region in the K. pneumoniae genome. Of note, CPS and LPS are not only two important virulence factors of K. pneumoniae but also antigenic targets for vaccine and phage-derived exopolysaccharide depolymerase, which is considered a novel approach for the treatment of CRKP infections (27). The diversity of CPS and LPS caused by the chromosomal recombination is posing a challenge to polysaccharide vaccine development. Thus, continued surveillance of CRKP populations remains of importance during vaccine development. Moreover, ST1764 (allelic profile 5-3-1-1-9-4-283), a three-locus variant of hypervirulent ST23 (allelic profile 2-1-1-1-9-4-12), is a regional ST entirely found in China so far and believed to be a hypervirulent clone (28–30). In some of our ongoing work, we have discovered 7 of 219 K. pneumoniae isolates, collected from pyogenic liver abscess patients, that belong to ST1764 (data not shown). The uniformity of epidemic geographical location of ST1764 and ST11 strains further confirms the recombination event.

Comandatore and coworkers proposed that the success of an emerging hybrid strain produced by a large recombination event depended on gene content in the exchanged genomic portion (31). Of note, we previously identified a clonal replacement in the CRKP-ST11 population over a 4-year period in a national key hospital, which indicated ST11-KL47, the previously predominant clone, was by degrees replaced by ST11-KL64 (17). It has been shown that the “hybrid” ST11-KL64 appeared to be more “successful” than its precursor, ST11-KL47, in the hospital. We compared the gene compositions in the exchanged genomic regions between ST11-KL64 and ST11-KL47. The results indicated that only genes involved in CPS and LPS biosynthesis and transposase-encoding genes in ISs were distinctly changed following the recombination. All these findings suggested that the obtained KL64 and/or O2 from the exchanged genomic donor portion was likely to be a critical factor conferring a survival advantage on the hybrid ST11-KL64 in response to external selection pressures (e.g., the immune response).

A previous study showed that among K. pneumoniae isolates causing pyogenic liver abscess, the O1 serotype was highly predominant, with a prevalence rate of approximately 90% (32). The O1 antigen contains two domains: d-galactan I and d-galactan II, while the O2 antigen is composed only of d-galactan I and is not recognized by anti-d-galactan II antiserum. Therefore, the d-galactan II structure is the difference between the LPS composition of K. pneumoniae serotypes O1 and O2. Previous studies indicated that the presence of d-galactan II was required for the resistance of K. pneumoniae to serum killing, and the d-galactan II-deficient mutant was less virulent than the parental strain in mouse intraperitoneal infection models (19, 33). Thus, d-galactan II is a crucial LPS composition in K. pneumoniae which enables the bacterium to exhibit a higher virulence potential. In this study, we found that although the ~154-kb region covering OL in CC1764 (except ST30) was recombined to the chromosome of ST11-KL47, the OL of the recombination offspring could only directly transform from OL101 into O2 (as with ST11-KL64) but not O1 (as with CC1764-KL64) due to the absence of d-galactan II-encoding *wbbY* and *wbbZ* genes in the ST11 chromosome. All these findings implied that the ST11-KL64 clone might not exhibit a similar virulence trait as ST1764 strains.

Nevertheless, we also found genetic diversity within the ST11-KL64 group. A ST11-KL64 isolate HB25-1 (18), identified in China, was found to have a homologous ~485-kb recombination region derived from ST147-KL64 strains (recombination II). Subsequently, we discovered that this recombination II event also produced a specific ST11-KL64 subclone which was significantly more infrequent than the predominant ST11-KL64 subclone produced by recombination I in China, despite showing the same KL64 and O2v1 in these two subclones. ST147-KL64 is a highly prevalent carbapenem-resistant clone in Asian countries such as India, Pakistan, and Thailand. The similarity of geographical distribution of ST147-KL64 and ST11-KL64 also provides support to the assumed recombination II event. However, the reason why the ST11-KL64 subclone produced by recombination II was not as prevalent as the subclone produced by recombination I remains to be elucidated.

There are certain limitations associated with our study. First, we could not find the explicit breakpoint of the recombination event, because there was no apparent difference existing in the large nucleotide fragments adjacent to the terminal of the region which contained a large number of SNPs. Therefore, we provided an approximate recombination region based on the SNP analysis and comparative genomic analysis. Second, a mechanism to explain the large-scale exchange of sequence observed in K. pneumoniae was not confirmed in the study. It was proposed that conjugation and transformation were efficient mechanisms of large-scale transfer of sequence in Streptococcus pneumoniae and Streptococcus agalactiae, but how it has occurred in Gram-negative bacteria remains a difficult issue (7, 34, 35). Third, we could not collect the ST11-KL64 strain produced by recombination II during the study period. Therefore, we were unable to explore the differences in microbiological characteristics between these two subclones.

Taken together, our findings underlined the role of recombination in the evolution of clinical strains of widely distributed KPC-associated ST11 K. pneumoniae in China. Understanding the evolutionary genomic history of the microbe is a critical approach to gain new insight into its success as a pathogen, as we have done here. This information will assist our efforts to develop diagnostics, therapeutics, and vaccines against infections caused by CRKP. Notably, CPS and LPS of K. pneumoniae contribute to the evasion of the innate host defense and thus are considered crucial for survival in the host. The switching of CPS and LPS in the newly emerging hybrid ST11 descendant altered antigens on the surface of the bacteria and made the production of a polysaccharide-based vaccine (such as phage-derived exopolysaccharide depolymerase) against K. pneumoniae infection more complicated, posing a substantial threat to health care networks.

## MATERIALS AND METHODS

### Isolates and whole-genome sequencing.

We previously collected 150 genomes of ST11 carbapenem-resistant K. pneumoniae (KL47, *n* = 71; KL64, *n* = 77; KL105, *n* = 1; KL103, *n* = 1) from patients with a blood culture positive for K. pneumoniae and a clinical course consistent with bloodstream infection during 2012 to 2017 in the First Affiliated Hospital of Zhejiang University in China (17). One ST1764-KL64 carbapenem-susceptible K. pneumoniae strain (KP41076) was identified in the hospital and sequenced by using an Illumina Hiseq2,500 instrument (Illumina) with 2 × 125-bp paired-end libraries. We performed *de novo* assembly of the short-read data by using Unicycler 0.4.0 (36). The ST of the isolate was acquired by MLST (https://cge.food.dtu.dk/services/MLST/). We annotated the assembly by using the RAST server (https://rast.nmpdr.org) (37). The 151 genomes of the above strains and 34 downloaded genomes of K. pneumoniae strains isolated from China, which belonged to ST11 (KL47, *n* = 13; KL64, *n* = 21), were added to the genome group for genomic analysis (see Table S1 in the supplemental material). Moreover, we analyzed 22,600 K. pneumoniae genomes among global isolates from PATRIC (38) for selecting the STs with the KL64 genetic locus. We found KL64 could be detected in the genomes of ST1764 (allelic profile 5-3-1-1-9-4-283), ST3685 (5-3-1-232-9-4-283), ST1764-1LV (5-3-1-1-9-4-683), ST30 (5-3-1-1-9-4-26), ST505 (7-1-5-1-1-1-84), ST147 (3-4-6-1-7-4-38), and ST11 (3-3-1-1-1-1-4). Using the stringent criteria, where all members share identical alleles at 6 of the 7 loci with at least one other member of the group, we defined ST1764, ST3685, ST1764-1LV, and ST30 as a clonal complex, CC1764. Ten genomes of CC1764 (ST1764, *n* = 4; ST3685, *n* = 1, ST1764-1LV, *n* = 1; ST30, *n* = 4), 8 ST505, and 45 ST147 were downloaded and used for genomic analysis. The detailed data of all 248 isolates are provided in Table S1.

Furthermore, 1,150 genomes of ST11 K. pneumoniae strains isolated from China were downloaded from PATRIC and screened for selecting ST11-KL64 genomes. As a result, 621 Chinese ST11-KL64 K. pneumoniae genomes were detected and used in the phylogenetic analysis to investigate the prevalence of the specific ST11-KL64 subclone. The detailed data of these 621 isolates are shown in Table S2.

### Genomic analysis.

Antimicrobial resistance genes and virulence genes were detected using Abricate (https://github.com/tseemann/abricate) with gene databases ResFinder (39) and VFDB (40), respectively. We identified K-type, O-type, and multilocus sequence typing by using Kleborate and Kaptive (41, 42). Insertion sequences were identified using the ISsaga (http://issaga.biotoul.fr/issaga_index.php) and the IS Finder database (https://www-is.biotoul.fr/) (43, 44). The complete nucleotide sequence of an ICE was identified by ICEfinder (https://db-mml.sjtu.edu.cn/ICEfinder/) with manual modification (45). Prophages were detected by using VRprofile (https://tool2-mml.sjtu.edu.cn/VRprofile/). Multiple genome sequence alignments and comparison analysis were then performed with Mauve (46). Sequence comparisons were performed using BLASTn v2.4.0 (47) and visualized using Easyfig v2.2.3 (48).

### Phylogenetic analysis.

Snippy v4.6.0 (https://github.com/tseemann/snippy) was used to perform reference-based mapping and identify SNPs in the core genome, with the KP47434 genome (GenBank accession no. QURI00000000) or the HB25-1 genome (GenBank accession no. CP039524, CP039525, CP039526) used as references. An SNP alignment was then extracted from the core genome alignment using snp-sites v2.5.1 (49), and a phylogenetic tree was constructed using the core SNPs with Fasttree 2.1 with default parameters (50). The phylogenetic tree was visualized using the Interactive Tree of Life software (iTOL v.3) software (51). The minimum spanning trees (MSTs) based on SNP data were generated using PHYLOViZ v2.0 (52).

### Data availability.

The whole-genome sequences of all collected K. pneumoniae isolates have been deposited in the GenBank database under BioProject numbers PRJNA588307 and PRJNA798548. The data that support the findings of this study are available and accession numbers of the genome sequences are listed in Table S1 and Table S2.
