# Supplementary material for: Recombination Drives Evolution of Carbapenem-Resistant Klebsiella pneumoniae Sequence Type 11 KL47 to KL64 in China
Source: Microbiol Spectr. 2023 Jan 9;11(1):e01107-22. doi: 10.1128/spectrum.01107-22 (PMC9927301; doi:10.1128/spectrum.01107-22)
Supplement: Supplemental file 5 — Fig. S1 to S4. Download spectrum.01107-22-s0005.pdf, PDF file, 1.4 MB [file spectrum.01107-22-s0005.pdf]

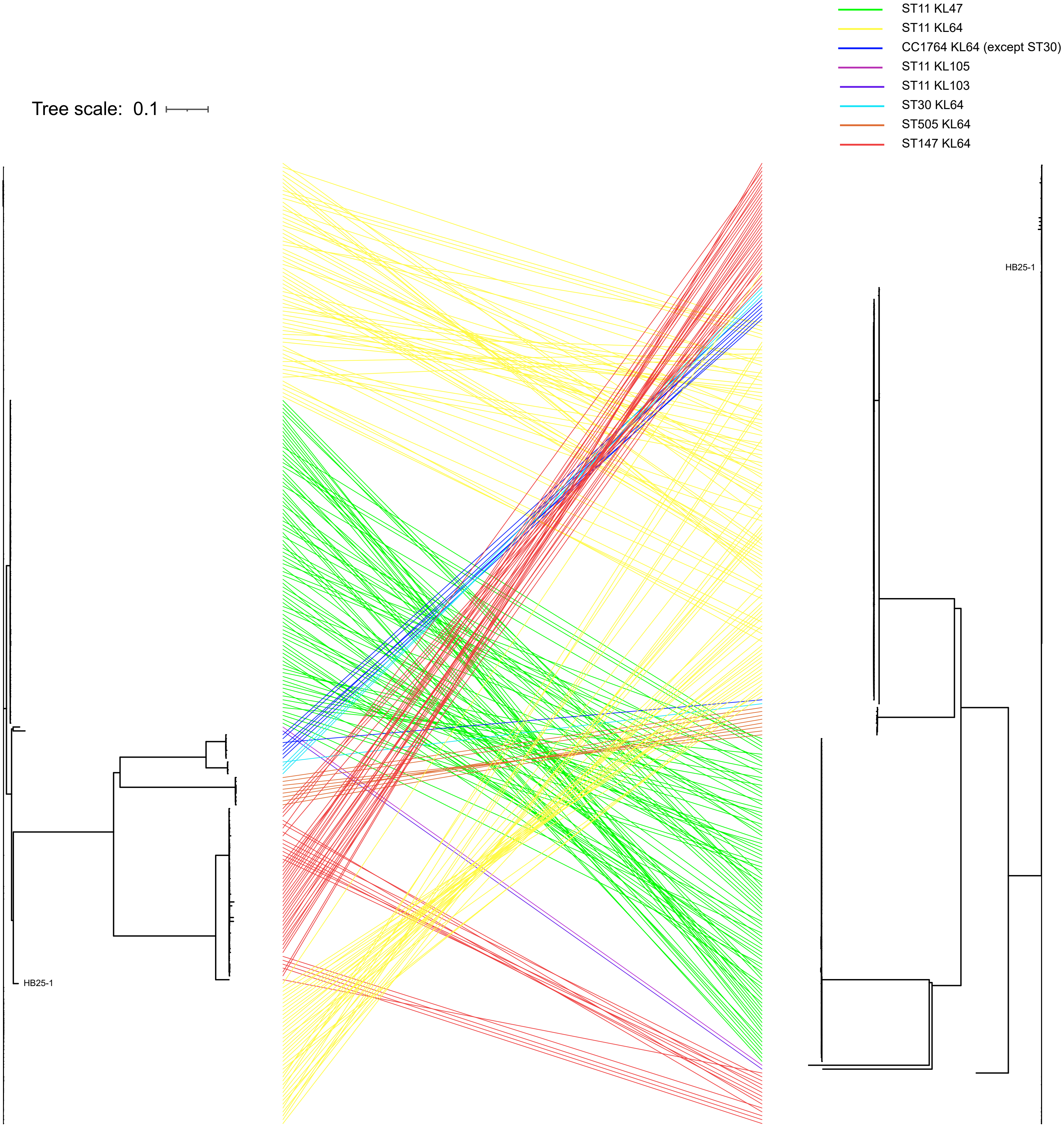

Figure S1. A co-phylogenetic plot of 248 *Klebsiella pneumoniae* strains based on genomic core SNPs (left) and core SNPs located in the ~154-kb region (right).

The same strain on the two phylogenetic trees is connected by lines with different colours according to STs and KLs.

The co-phylogenetic plot was created by using “cophylo” R library.

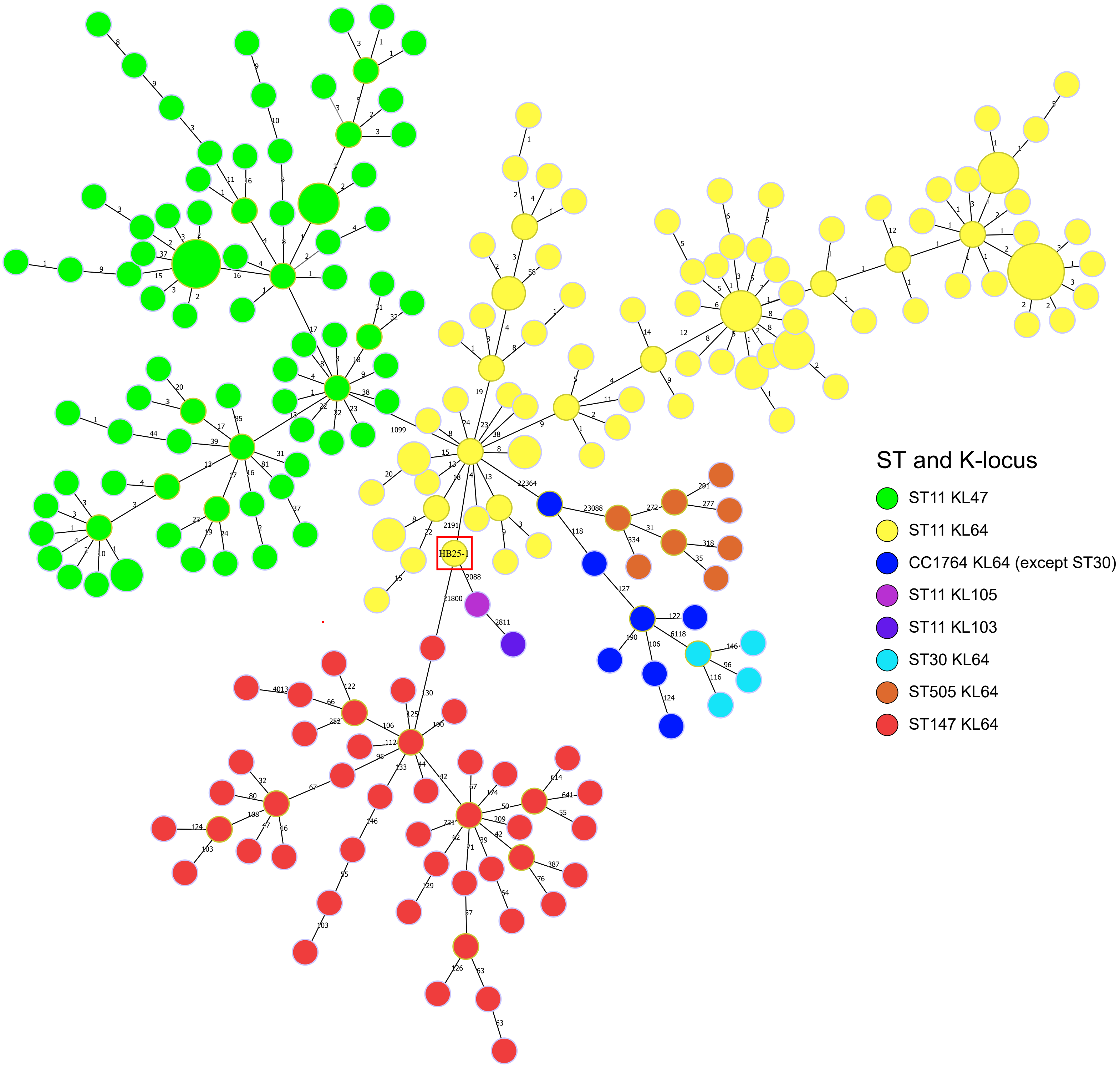

Figure S2. The minimum spanning tree (MST) of 248 *Klebsiella pneumoniae* strains based on genomic core SNPs. Strains are represented by circles, and the size of the circle is proportional to the number of isolates with identical SNPs. The colours of the circle were assigned according to STs and KLs. The numbers on the connecting lines illustrate the number of SNPs in a pairwise comparison.

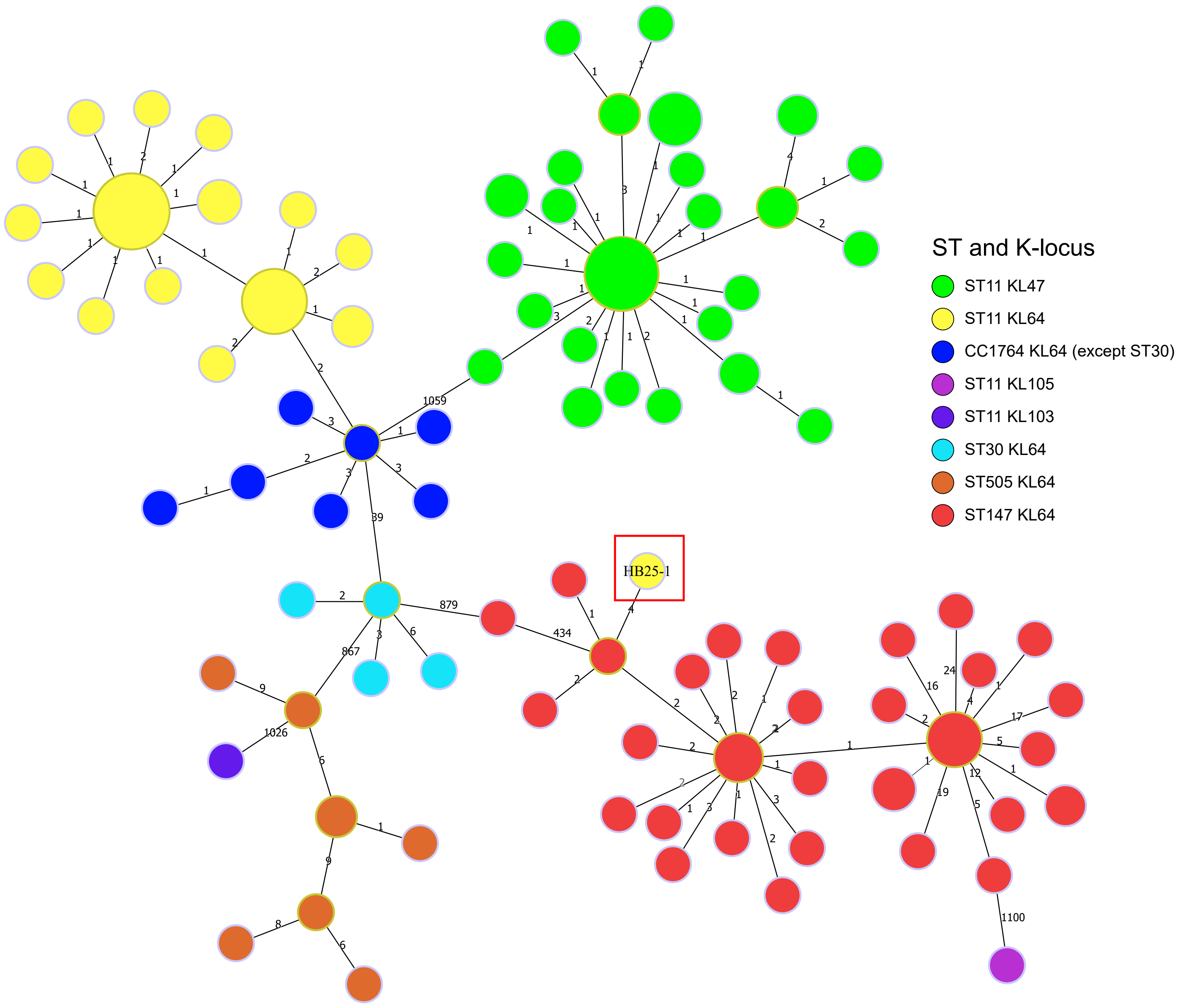

Figure S3. The minimum spanning tree (MST) of 248 *Klebsiella pneumoniae* strains based on core SNPs located in the ~154-kb region. Strains are represented by circles, and the size of the circle is proportional to the number of isolates with identical SNPs. The colours of the circle were assigned according to STs and KLs. The numbers on the connecting lines illustrate the number of SNPs in a pairwise comparison.

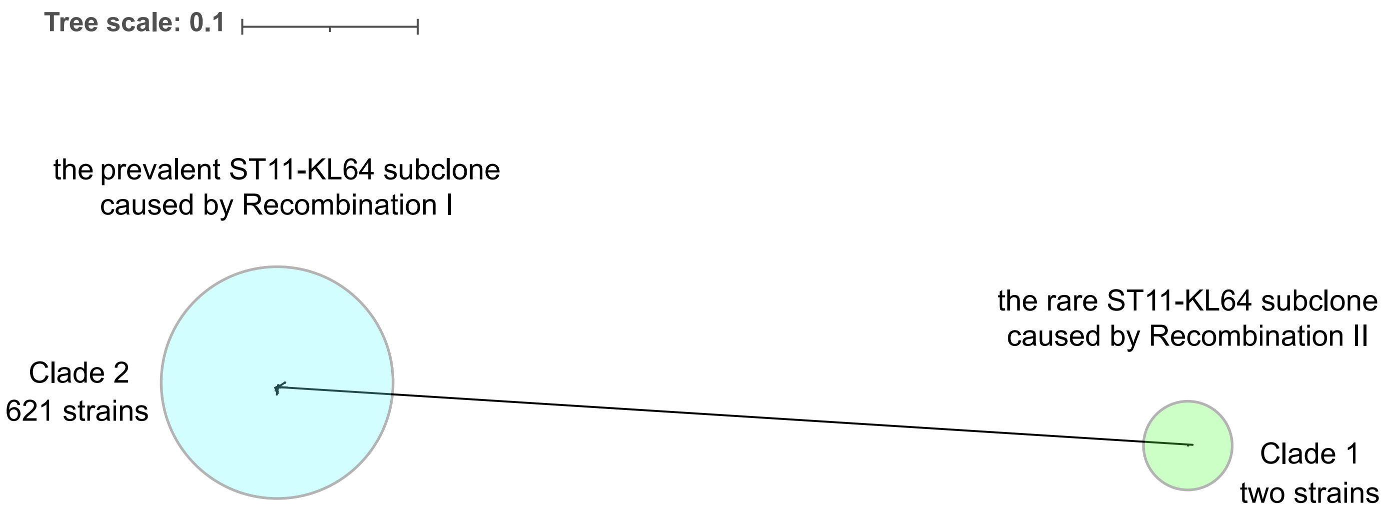

Figure S4. Phylogenetic analysis of 623 genomes of ST11-KL64 *K. pneumoniae* strains isolated from China (including KP_47434 and HB25-1) on the core SNPs located in the ~485-kb region. Only one of 621 genomes was gathered with strain HB25-1 in Clade 1, while other 620 genomes were gathered with KP_47434 in Clade 2.
